# Supplementary material for: Integrating causal inference and machine learning to quantify climate-malaria relationships: Evidence of temperature and rainfall thresholds from Colombian municipalities
Source: PLOS Glob Public Health. 2026 Feb 5;6(2):e0005925. doi: 10.1371/journal.pgph.0005925 (PMC12875494; doi:10.1371/journal.pgph.0005925)
Supplement: S1 Fig — (DOCX) [file pgph.0005925.s001.docx]

S1_Fig

Records with spatial thinning to 25 km^2^ for the *Anopheles* species included in the analysis of vector co-occurrence.


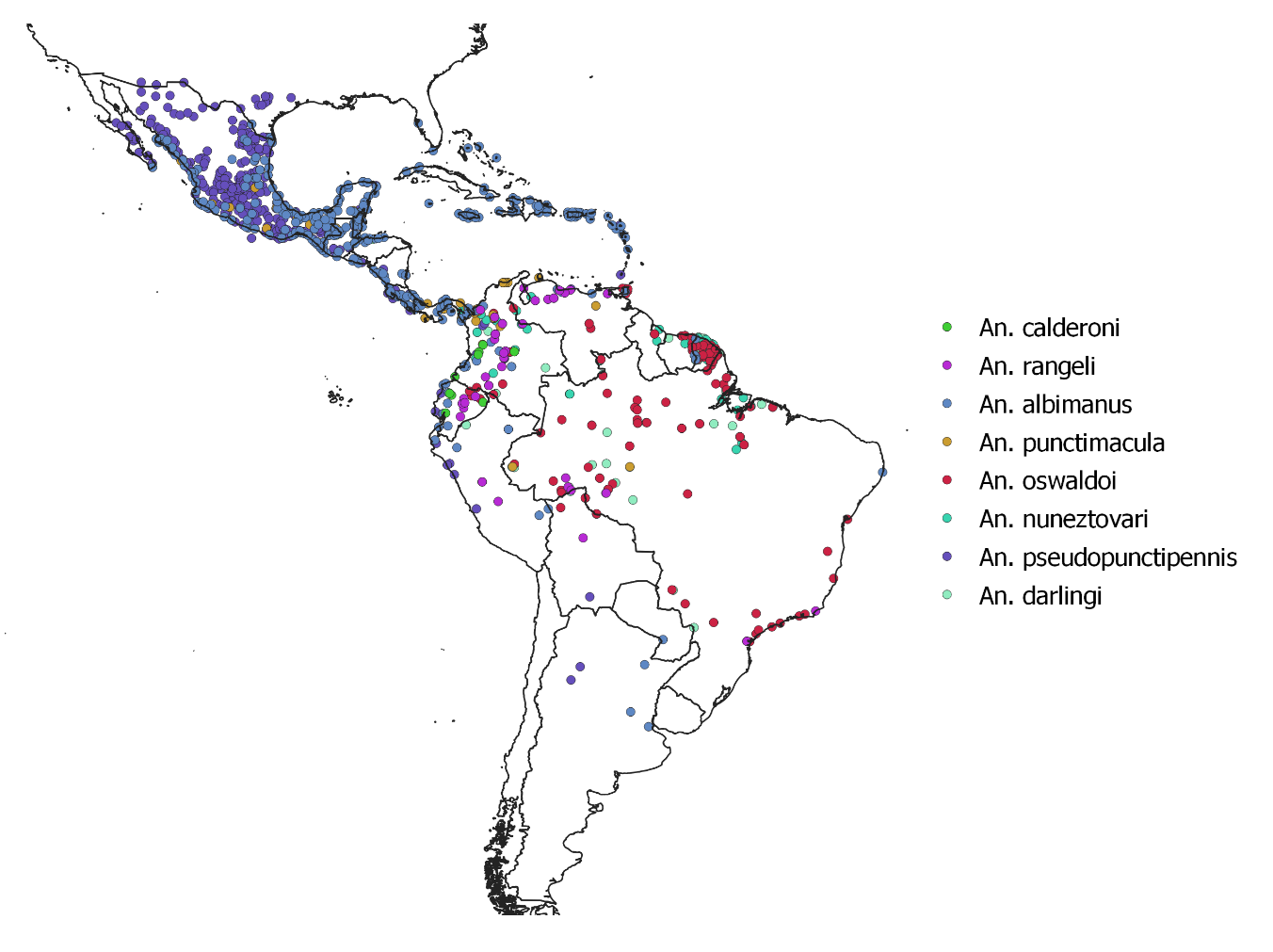


Note: The map was created using QGIS software, with the basemap shapefile (https://www.naturalearthdata.com/http//www.naturalearthdata.com/download/110m/physical/ne_110m_land.zip) sourced from Natural Earth, an openly available resource (https://www.naturalearthdata.com/). The terms of use for the base shapefile are compatible with public domain standards (https://www.naturalearthdata.com/about/terms-of-use/).
