# Supplementary material for: Integrating causal inference and machine learning to quantify climate-malaria relationships: Evidence of temperature and rainfall thresholds from Colombian municipalities
Source: PLOS Glob Public Health. 2026 Feb 5;6(2):e0005925. doi: 10.1371/journal.pgph.0005925 (PMC12875494; doi:10.1371/journal.pgph.0005925)
Supplement: S1 Text — (DOCX) [file pgph.0005925.s003.docx]

S1_Text

Technical details of the Causal Inference Analysis, Machine Learning Implementation, and Robustness and Sensitivity Tests.

Causal Inference Analysis

We emulated two experimental designs to estimate with observational data, the effect of rainfall and temperature on the SIR of malaria. For this purpose, we developed a Directed Acyclic Graph (DAG) to evidence our prior understanding of the relationships between the variables in our epidemiologic analysis and explicitly state our assumptions [37]. The DAGs incorporated rainfall and temperature as the exposure variables, and the SIR of malaria as the outcome variable.

The next figure corresponds to the DAGs for the effect of rainfall (a) and temperature (b) on malaria. Note that the black arrow indicates the causal association of interest. In the figure, SST = Sea surface temperature indices, EVI = Enhanced vegetation index, MPI = Multidimensional poverty index. The asterisk on the node SST indicates the correlation among these indices. Municipality = Descriptor variable for each municipality. Municipality-Year = Descriptor variable for each municipality in each year. Municipality-Year-Month = Descriptor variable for each municipality in each year and each month.


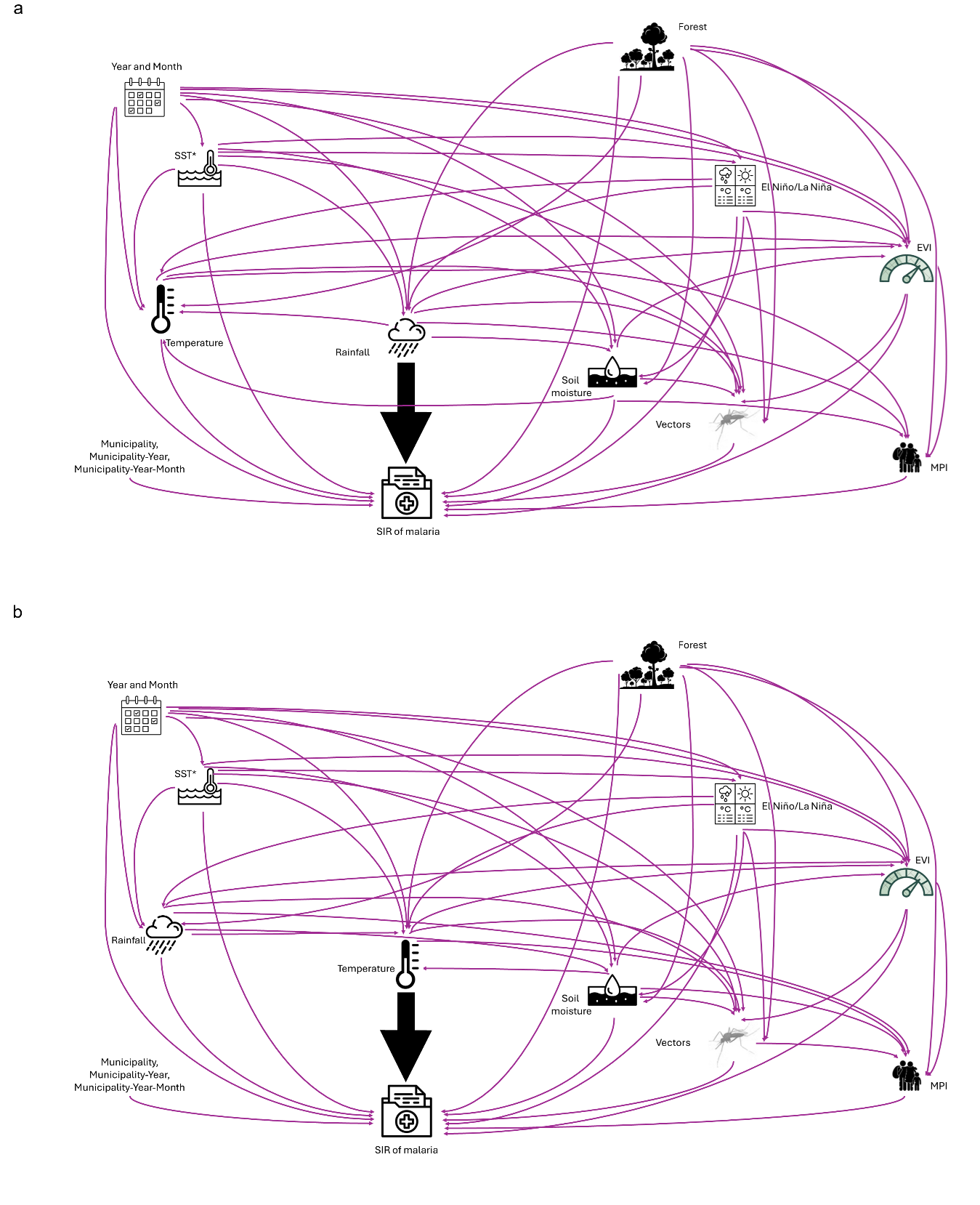


Note: The figure was created using open-source images from Openclipart (https://openclipart.org), which are released into the public domain under the CC0 1.0 license 202 (https://openclipart.org/faq).

The causal interpretation of our findings regarding the effect of rainfall and temperature on the SIR of malaria depends on meeting several key assumptions [38–40]: a) No unmeasured confounding: After accounting for all potential confounding variables, the outcome remains independent of the exposure variable. b) Treatment definition consistency: The exposure measure is clearly defined so that variations within its definition would not alter the outcome, and no interference between units occurs. c) Overlap assumption: Each exposure level has a non-zero probability of being observed across all covariate combinations within the municipalities under study. d) Absence of measurement bias: No significant errors in measurement exist that would introduce substantial bias into the analysis. e) Correct model specification: The model appropriately captures all relevant non-linear relationships and interaction terms.

We used the package ggdag (version 0.2.10) [41] in R to identify the correct adjustment to emulate the experimental design and estimate the effect thereby avoiding the introduction of bias.

Machine Learning Implementation

- Data Preprocessing and Feature Engineering

Prior to causal estimation, comprehensive data preprocessing was conducted to optimize model performance and ensure methodological rigor. Temporal variables were transformed to capture cyclical patterns, with the year variable normalized by subtracting the baseline year (2007) and monthly seasonality encoded using sine and cosine transformations to preserve the cyclical nature of seasonal data, where December naturally connects to January rather than being treated as distant endpoints in a linear sequence.

Co-variables were converted to binary values using median-based thresholds to reduce dimensionality and enhance model stability. The exposure variables were constrained to the ranges of 0.01 – 60 mm (rainfall) and 15 – 30 °C (temperature) to ensure the overlap assumption, eliminating extreme outliers and guaranteeing biological plausibility. At the same time, a complete case analysis was implemented to handle missing data.

- Targeted Maximum Likelihood Estimation Framework

Causal effects were estimated using a modified Targeted Maximum Likelihood Estimation (TMLE) approach implemented through the causal-curve package's TMLE_Regressor class [42], which incorporates machine learning techniques through gradient boosting and produces confidence intervals.

The TMLE framework addresses confounding bias in observational studies by combining outcome regression (Q-model) and treatment mechanism modeling (G-model) within a doubly robust estimation procedure. This approach provides consistent estimates even when one of the two models is misspecified, making it particularly suitable for complex epidemiological data with potential unmeasured confounding.

- Gradient Boosting Implementation and Hyperparameter Configuration

The TMLE_Regressor was configured with 1,500 gradient boosting estimators to ensure sufficient model complexity for capturing non-linear relationships between rainfall and temperature and the SIR of malaria. A conservative learning rate of 0.0001 was implemented to prevent overfitting and improve convergence stability, while maximum tree depth was limited to 5 levels to maintain interpretability and reduce variance. Random seed initialization ensured reproducibility of results across model runs, while verbose output was enabled to monitor convergence diagnostics during training.

Note that the TMLE framework provides double robustness and asymptotic efficiency, but its finite-sample performance depends on the accuracy of the nuisance function estimates—specifically, the outcome regression (Q-model) and exposure mechanism (G-model). To flexibly model these nuisance components, we employed a gradient boosting algorithm whose hyperparameters were set a priori based on theoretical and empirical considerations from the targeted learning literature.

In that sense, we used a large number of estimators (n_estimators = 1,500) combined with a conservative learning rate (1e−4) to allow the boosting algorithm to learn complex, nonlinear patterns gradually and to minimize the risk of overfitting. The maximum tree depth was restricted to maintain interpretability and to control variance. Although the causal-curve package does not implement an automatic cross-validation or cross-validated TMLE procedure, the theoretical properties of TMLE provide intrinsic robustness to moderate model misspecification, as long as at least one nuisance model is well estimated. Our configuration follows standard practice for environmental and epidemiological applications where stability and smooth convergence are prioritized over aggressive hyperparameter tuning [57].

- Causal Exposure-Response Curve Estimation and Inference

The causal exposure-response curve estimation process involved systematic evaluation across the full treatment range to identify exposure levels and quantify causal effects. Confidence intervals were calculated at the 95% level using the calculate_CDRC method of the Python package causal-curve, providing uncertainty quantification around point estimates through bootstrap-based inference procedures. The resulting causal curves enabled identification of treatment ranges that causally produce optimal outcomes while accounting for the complex confounding structure inherent in observational environmental health data.

We estimated 95% uncertainty intervals around the causal exposure–response curves to quantify sampling variability in the estimated effects. These intervals were constructed using the variance estimator implemented within the TMLE_Regressor framework: specifically, the standard errors are derived from the estimated influence function of the targeted estimator and, where indicated, complemented by a nonparametric bootstrap to validate finite-sample behavior. The reported bands are pointwise 95% intervals (that is, each exposure value has its own interval), rather than simultaneous bands across the whole curve.

Robustness and Sensitivity Tests

- Negative Control Assessment for Residual Confounding

To evaluate the robustness of our causal exposure-response estimates to the presence of residual confounding bias, we implemented the negative control method proposed by Flanders et al. [43]. This approach leverages the temporal ordering principle that causes must precede their effects to detect unmeasured confounding. The method relies on an indicator variable that satisfies two key characteristics: first, it must be conditionally independent of the outcome when the model is correctly specified (i.e., it cannot cause the outcome), and second, it should be associated with both the exposure of interest and any unmeasured confounders.

In our time-series analysis of the effect of rainfall and temperature on the SIR of malaria, we used advanced measurements (rainfall in t+1 and temperature in t+1) from the subsequent time period as the negative control indicator, following the rationale that future values cannot have caused past malaria cases but would share common unmeasured confounders with exposures.

After obtaining causal exposure-response curves, we generated predicted malaria incidence rates through interpolation of the estimated causal relationship. We then fitted a Gamma generalized linear model with the observed outcome as the dependent variable and both the predicted outcome (from our causal model) and the negative control indicator (rainfall in t+1 and temperature in t+1) as predictors.

The statistical significance of the coefficient for the negative control variable serves as a diagnostic test for model misspecification. A statistically significant association between the future indicators and past SIR of malaria would suggest the presence of residual confounding bias. Conversely, a non-significant coefficient supports the validity of our causal model specification and the adequacy of confounder control. Note that the negative control assessment for residual confounding was run independently for each outcome.

- E-Value Sensitivity Analysis Implementation

To assess the sensitivity of our causal estimates to potential unmeasured confounding, we conducted the E-value sensitivity analysis [44] using the EValue R package (version 2.0.0). The E-value quantifies the minimum strength of association, on the risk ratio scale, that an unmeasured confounder would need to have with both the treatment and outcome to fully explain away the observed causal effect.

Following our analysis, we calculated Cohen's d as the standardized effect size by dividing the causal effect difference between maximum and minimum exposures of rainfall and temperature by the standard deviation of the outcome variable. We exported the necessary parameters from Python to R, including the Cohen's d estimate, its standard error, and the confidence interval limits to enable comprehensive E-value calculations.

A higher E-value indicates greater tolerance to unmeasured confounding, as they require stronger confounding associations to nullify the observed effect. For example, in our study of the effect of temperature on the SIR of malaria, within the range of 15 to 30 °C, an E-value of 1.5 means that an unobserved confounder would have to: 1) increase the SIR by 50% when present, and 2) be 50% more frequent among observations at 30 °C than among those at 15 °C.

We interpreted the E-value in the context of plausible confounding scenarios in malaria epidemiology, considering whether unmeasured factors could realistically achieve the magnitude of associations required to eliminate our estimated causal effects. This sensitivity analysis provides a quantitative framework for evaluating the credibility of our causal inferences under realistic assumptions about potential unmeasured confounders.
