# Supplementary material for: Integrating causal inference and machine learning to quantify climate-malaria relationships: Evidence of temperature and rainfall thresholds from Colombian municipalities
Source: PLOS Glob Public Health. 2026 Feb 5;6(2):e0005925. doi: 10.1371/journal.pgph.0005925 (PMC12875494; doi:10.1371/journal.pgph.0005925)
Supplement: Attachment — Submitted filename: Response to Reviewers.docx [file pgph.0005925.s005.docx]

Journal Requirements:

1. Please send a completed 'Competing Interests' statement, including any COIs declared by your co-authors. If you have no competing interests to declare, please state "The authors have declared that no competing interests exist". Otherwise please declare all competing interests beginning with the statement "I have read the journal's policy and the authors of this manuscript have the following competing interests:"

R: We appreciate the comment, and we have rewritten the competing interests’ statement (line 539).

2. Some material included in your submission may be copyrighted. According to PLOS’s copyright policy, authors who use figures or other material (e.g., graphics, clipart, maps) from another author or copyright holder must demonstrate or obtain permission to publish this material under the Creative Commons Attribution 4.0 International (CC BY 4.0) License used by PLOS journals. Please closely review the details of PLOS’s copyright requirements here: PLOS Licenses and Copyright. If you need to request permissions from a copyright holder, you may use PLOS's Copyright Content Permission form.

Please respond directly to this email or email the journal office and provide any known details concerning your material's license terms and permissions required for reuse, even if you have not yet obtained copyright permissions or are unsure of your material's copyright compatibility.

Potential Copyright Issues:

Figure 2 and Supplement 1: please (a) provide a direct link to the base layer of the map (i.e., the country or region border shape) and ensure this is also included in the figure legend; and (b) provide a link to the terms of use / license information for the base layer image or shapefile. We cannot publish proprietary or copyrighted maps (e.g. Google Maps, Mapquest) and the terms of use for your map base layer must be compatible with our CC-BY 4.0 license.

Note: if you created the map in a software program like R or ArcGIS, please locate and indicate the source of the basemap shapefile onto which data has been plotted.

If your map was obtained from a copyrighted source please amend the figure so that the base map used is from an openly available source. Alternatively, please provide explicit written permission from the copyright holder granting you the right to publish the material under our CC-BY 4.0 license.

Please note that the following CC BY licenses are compatible with PLOS license: CC BY 4.0, CC BY 2.0 and CC BY 3.0, meanwhile such licenses as CC BY-ND 3.0 and others are not compatible due to additional restrictions.

If you are unsure whether you can use a map or not, please do reach out and we will be able to help you. The following websites are good examples of where you can source open access or public domain maps:

* U.S. Geological Survey (USGS) - All maps are in the public domain. (<http://www.usgs.gov>)

* PlaniGlobe - All maps are published under a Creative Commons license so please cite “PlaniGlobe, http://www.planiglobe.com, CC BY 2.0” in the image credit after the caption. (<http://www.planiglobe.com/?lang=enl>)

* Natural Earth - All maps are public domain. (<http://www.naturalearthdata.com/about/terms-of-use/>)

R: We appreciate the comment, and we have modified the legend of Figures 1 and 2, and Supplement 1, to clarify that the base maps used are from an openly available source, and we included the corresponding webpages of the sources.

3. If the reviewer comments include a recommendation to cite specific previously published works, please review and evaluate these publications to determine whether they are relevant and should be cited. There is no requirement to cite these works unless the editor has indicated otherwise.

########################

Reviewer #1:

- I recommend writing '1600 meters' instead of '1600m'.

R: We appreciate the comment, and we have made the change (line 15)

- The author fails to motivate the paper. Why is it worth it, the research? How can it be useful for public health policy in Colombia?

R: We accepted the comment, and we have included a couple of new paragraphs in the Introduction section to illustrate why the research is worth, and how the research can be useful for public health policy in Colombia (lines 79-97)

- Before estimating the causal model (temperature vs. malaria), I recommend including a spatial and temporal permutation model to localize the cluster. Neighboring municipalities

R: Thank you for your valuable observation. We acknowledge the importance of incorporating spatial and temporal analysis to account for the shared climatic and epidemiological characteristics among neighboring municipalities. To address this, we have integrated findings from our previous study (Tapias-Rivera & Gutiérrez, 2023) published in Acta Tropica, where we identified clusters of high malaria incidence in Colombia using spatial autocorrelation indices. This study localized malaria clusters in regions such as the Amazon, Pacific, and Urabá-Bajo Cauca-Alto Sinú, highlighting the influence of environmental and socio-economic variables. By leveraging these findings, here we are providing a causal inference framework for estimating the form of the relationship between climate variables and malaria incidence (lines 72-78).

- The authors could include an isotherm and isometric map of Colombia to better illustrate the geographical distribution of the results.

R: We accepted the comment, and we have included Figure 2, with the isomaps of temperature and rainfall (lines 275-286).

- The link for the replication codes should be at the end of the document, not in the methods section.

R: We appreciate the comment, and we have moved the paragraph to the end of the document (lines 543-548).

- The discussion section is missing. How do these results compare to previous literature? How does the paper fill the gap in existing research?

R: We appreciate the comment, and we have rewritten the Discussion section (lines 360-393) to compare our results with previous literature.

########################

Reviewer #2:

The manuscript titled “Integrating Causal Inference and Machine Learning to Quantify Climate–Malaria Relationships: Evidence of Temperature and Rainfall Thresholds from Colombian Municipalities” presents an ambitious and technically sophisticated ecological study that combines causal inference methods with machine learning to estimate the effects of temperature and rainfall on malaria incidence in Colombia. The study’s scope aligns with the objectives of PLOS Global Public Health, particularly its emphasis on methodological innovation and data-driven public health insights for climate-sensitive diseases.

Overall Assessment:

The study is methodologically rigorous, conceptually strong, and addresses an important global health question linking climate variability to malaria transmission dynamics. The combination of Directed Acyclic Graphs (DAGs) with Targeted Maximum Likelihood Estimation (TMLE) represents a cutting-edge analytical framework rarely applied in environmental epidemiology. The dataset is extensive, covering 969 municipalities over 17 years, which provides substantial statistical power for causal modeling. The conclusions drawn are largely consistent with the data presented and contribute meaningfully to understanding threshold-based climate–malaria relationships.

1. Scientific and Methodological Rigor

The research design is robust and transparent. The use of DAGs to guide variable selection and control for confounding is methodologically sound, and the TMLE framework is appropriately chosen to estimate causal effects in complex observational datasets. The paper demonstrates thoughtful consideration of confounding, model specification, and sensitivity analyses through E-values and negative control tests.

However, a few points merit clarification:

• The exclusion of certain variables (e.g., MPI) to prevent collider bias is justified but could be more explicitly linked to the DAG interpretation for readers unfamiliar with causal reasoning.

R: We appreciate the comment, and we have rewritten the paragraph to explicitly link the justification to the DAG interpretation for readers unfamiliar with causal reasoning (lines 294-302)

• The negative control findings indicate residual confounding bias (p < 0.05). While transparently reported, the implications of this residual bias for causal interpretation should be discussed in greater depth.

R: We accepted the comment, and we have modified the text (lines 415-441) to clarify the implications of this residual confounding bias for the causal interpretation.

• The assumptions of data independence and the decision not to model municipality-level clustering deserve further methodological justification, as repeated measures within municipalities likely violate independence assumptions.

R: We accepted the comment, and we have rewritten the paragraph to explicitly recognize this limitation (lines 410-414), and justify why we cannot currently incorporate the hierarchical structure of the data (lines 491-499).

Overall, the analytical framework is technically strong, but the authors should provide a clearer explanation of how these methodological limitations might influence the robustness of their causal estimates.

2. Statistical Analysis

The statistical analysis is advanced and implemented with rigor. The TMLE - gradient boosting integration, exposure-response curve estimation, and E-value sensitivity analyses demonstrate high analytical competency. The approach is well-suited for modeling non-linear relationships and estimating causal effects from high-dimensional data.

Nevertheless, the manuscript could benefit from:

• Simplified descriptions of the statistical models to improve accessibility for readers who may not be familiar with causal machine learning.

R: We accepted the comment, and we have rewritten the section of Methods (lines 197-259) to make it accessible for readers who may not be familiar with causal machine learning, and we have moved the technical details to supplementary material 3.

• Clarification on hyperparameter tuning procedures (e.g., justification for 1,500 estimators and learning rate selection) and whether cross-validation or out-of-sample validation was applied to mitigate overfitting.

R: We appreciate the comment, and we have included a justification of hyperparameters to avoid overfitting in the supplementary material 3 (pg. 4, paragraph 3 and pg. 5, paragraph 1).

• A clearer presentation of uncertainty intervals and their interpretation in the context of causal inference rather than traditional regression.

R: We accepted the comment, and we have included a clarification about the mechanism to estimate the uncertainty intervals in the supplementary material 3 (pg. 6, paragraph 3), and we included a couple of new paragraphs in the Discussion to clarify the interpretation of confidence intervals in the context of causal inference (lines 405-422)

Despite these areas for improvement, the statistical analysis is appropriately conducted and sufficiently rigorous to support the stated conclusions.

3. Ethical and Publication Standards

The authors have stated that the study was approved by the Universidad de Santander Bioethics Committee and that only anonymized, aggregated surveillance data were used. There are no apparent ethical concerns regarding participant privacy or data integrity. The study adheres to STROBE reporting standards, which strengthens transparency and replicability. I found no evidence of dual publication or plagiarism.

All data sources and code repositories are clearly cited and publicly accessible, which aligns with PLOS’s open science policies.

4. Presentation and Language Quality

The manuscript is written in standard scientific English and is generally intelligible. However, it is overly dense and highly technical, particularly in the Methods and Results sections. Long sentences, heavy use of jargon, and algorithmic detail (e.g., references to “verbose outputs” and “gradient boosting hyperparameters”) make some sections difficult to follow for the journal’s broader public health audience.

The manuscript would benefit from:

• Language editing to improve readability, sentence flow, and paragraph structure.

• Condensation of repetitive methodological details (e.g., repeated mention of DAGs, confounding, and TMLE framework).

• Improved figure legends and summaries that allow independent interpretation of graphs and exposure-response curves.

R: We appreciate the comments, and in the new version of the manuscript, we have improved these stylistic issues.

Despite these stylistic issues, the text remains clear enough for reviewers to evaluate the science, and the English usage meets baseline journal standards.

5. Interpretation and Public Health Relevance

The findings that malaria incidence peaks at approximately 25 °C and 37 mm monthly rainfall are biologically plausible and consistent with previous literature. However, the discussion is heavily methodological and could better emphasize the public health significance of these results. The authors should explicitly describe how these climatic thresholds could be integrated into malaria early warning systems, inform vector control scheduling, or support climate adaptation planning in malaria-endemic regions. Connecting the technical results more directly to applied health security and surveillance planning would significantly enhance the paper’s impact.

R: We appreciate the comment, and we have included a new paragraph to emphasize the public health significance of our results (lines 500-510).

6. Recommendations for Improvement

1. Simplify technical sections to enhance accessibility to multidisciplinary readers.

2. Expand discussion on the implications of residual confounding and independence assumption violations.

3. Provide clearer linkage between climatic thresholds and practical malaria control strategies.

4. Condense repetitive text and streamline figures for clarity.

5. Consider professional language and structural editing before publication.

R: We appreciate the five recommendations, and the reviewer can see the specific response to each recommendation in the previous answers and confirm the changes in the new version of the manuscript and in the supplementary material 3.

Final Evaluation

• Scientific soundness: Strong

• Methodological rigor: High

• Statistical validity: Appropriate, though slightly overtechnical

• Ethical standards: Fully met

• Readability: Moderate; needs editing for clarity

• Suitability for publication in PLOS Global Public Health: Yes, pending minor to moderate revision for clarity and accessibility.
