# Supplementary material for: Integrating causal inference and machine learning to quantify climate-malaria relationships: Evidence of temperature and rainfall thresholds from Colombian municipalities
Source: PLOS Glob Public Health. 2026 Feb 5;6(2):e0005925. doi: 10.1371/journal.pgph.0005925 (PMC12875494; doi:10.1371/journal.pgph.0005925)
Supplement: Attachment — Submitted filename: Response to Reviewrs.docx [file pgph.0005925.s006.docx]

Review Comments to the Author

Please use the space provided to explain your answers to the questions above. You may also include additional comments for the author, including concerns about dual publication, research ethics, or publication ethics. (Please upload your review as an attachment if it exceeds 20,000 characters)

Reviewer #2: (No Response)

Reviewer #3: This manuscript presents a compelling approach to estimating the effects of rainfall and temperature on malaria incidence. The introduction is concise and effectively establishes the context. The methods and analyses appear robust and are clearly explained. I particularly appreciate the comprehensive explanation provided for each analysis. The results are succinct and well-articulated. While the discussion is somewhat lengthy and addresses numerous points, all are valid and interesting. I only have a few comments and suggestions for the author:

- Methods: On lines 111-112, please provide the reference for the STROBE guidelines utilized.

R: We accepted the suggestion and we have included the reference for the STROBE guidelines utilized (line 111)

- Methods: Lines 120-121, regarding the sentence ‘Furthermore, data from municipalities exceeding 1,600 meters in elevation were excluded, based on the National Health Institute's established cutoff for malaria transmission viability [14]’. Is this reference accurate? Did the National Health Institute establish this cutoff? Please clarify whether the National Health Institute is the appropriate source, or if another publication should be cited for this cutoff.

R: We appreciate the comment, the National Health Institute did not establish this cutoff, we included the correct publication for this cutoff, which is Rodríguez et al. (2011) (line 120-121)

- Discussion: I recommend including subheadings or sections within the discussion, as there are several important topics to address: the main results (temperature and rainfall gradients), the significance of using local data and models (a major strength of the paper), the methodological approach (machine learning, causal inference, etc.), and the limitations and challenges. Organizing the discussion in this way would enhance readability. This is a suggestion for the author, if it aligns with the journal's guidelines.

R: We accepted the suggestion and we have included subheadings in the discussion (lines 351-537)

- Discussion: As an additional suggestion, given the length of the discussion, I believe it would be valuable to elaborate on the relevance of these results and methods in the context of climate change. Although the primary focus of the paper is not climate change, including one or two sentences on how this research can inform climate change studies would be beneficial. The Intergovernmental Panel on Climate Change is currently preparing its next Assessment Report and is seeking relevant research, particularly studies incorporating local data from the Global South, such as this one. Furthermore, the discussion could be strengthened by addressing how climate change may influence the values obtained, especially considering that some studies suggest mosquitoes are expanding their altitudinal range as average temperatures rise. I encourage the author to comment on this aspect.

R: We accepted the suggestion and we have included a new subheading titled “4.4 Climate Change Implications and Altitudinal Range Expansion“ (lines 515-537)
